# Efficacy and safety of Bujing Yishi tablet for glaucoma with controlled IOP: study protocol for a multi-centre randomized controlled trial

**DOI:** 10.1186/s13063-020-04249-7

**Published:** 2020-05-25

**Authors:** Hongji Liu, Xiang Li, Zongduan Zhang, Jieping Zeng, Yan Dai, Chao Wang, Zhao Xie, Lin Cheng, Linru Cui

**Affiliations:** 1 Department of Ophthalmology, Mianyang Central Hospital, Mianyang, 621000 Sichuan China; 2 College of Ophthalmology, Chengdu University of Traditional Chinese Medicine, Chengdu, 610075 Sichuan China; 3 Department of Ophthalmology, Hospital of Chengdu University of Traditional Chinese Medicine, Chengdu, 610075 Sichuan China; 4 Department of Ophthalmology, The Affiliated Eye Hospital of Wenzhou Medical University, Wenzhou, 325027 Zhejiang China; 5 National Drug Clinical Trial Agency, Hospital of Chengdu University of Traditional Chinese Medicine, Chengdu, 610075 Sichuan China; 6 Department of Ophthalmology, Mianyang Hospital of Traditional Chinese Medicine, Mianyang, 621000 Sichuan China; 7 Department of Ophthalmology, The Second People’s Hospital of Chengdu PiDu District, Chengdu, 611733 Sichuan China; 8 Zhongshan Ophthalmic Center, Sun Yat-sen University, Guangzhou, 510275 Guangdong China

**Keywords:** Bujing Yishi tablet, Traditional Chinese medicine, Visual function protection, Glaucoma, Randomized controlled trial

## Abstract

**Background:**

As an irreversible, intractable disease with vision loss, glaucoma leads to permanent and progressive damage of visual function. Lowering high intraocular pressure (HIOP) is the first choice for treating glaucoma; however, the control of HIOP is not enough to prevent progressive vison loss. Currently, the therapies to treat glaucoma with controlled IOP (GPCI) are unsatisfactory. Chinese medicine is effective for improving visual function in patients with GPCI. Bujing Yishi tablets (BJYSP) have been the standard preparation for treating GPCI in our hospital for decades. However, no rigorous randomized controlled clinical studies have investigated its effects and safety.

**Methods:**

This study will be a 6-month, multicenter, stratified trial following a prospective, randomized, open-label, blinded endpoint (PROBE) protocol. A total of 216 eligible GPCI patients aged 18–75 years will be stratified according to the early, moderate, and advanced stages of glaucoma. After stratifying, the participants will be randomly assigned to the BJYSP group or control group at a ratio of 1:1. Following randomization, participants in the BJYSP group and control group will receive BJYSP and mecobalamin tablets, respectively, for the same 6-month period. The primary outcomes will include the best-corrected visual acuity (BCVA), visual field assessment, visual evoked potential (VEP) test, and Heidelberg retina tomography II (HRT II); the secondary outcomes will include intraocular pressure (IOP) and Traditional Chinese medicine (TCM) clinical symptom scales. The primary and secondary outcomes will be measured at baseline and 8, 16, and 24 weeks thereafter. Safety assessments will also be evaluated at baseline and 12 and 24 weeks thereafter.

**Discussion:**

This study will be a standardized, scientific, clinical trial designed to evaluate the therapeutic effects and safety of BJYSP as a novel therapeutic strategy for improving visual function in patients with GPCI.

**Trial registration:**

Chinese Clinical Trial Registry, ChiCTR1800016431. Registered on 1 June 2018.

## Administrative information

Note: the numbers in curly brackets in this protocol refer to SPIRIT checklist item numbers. The order of the items has been modified to group similar items (see http://www.equator-network.org/reporting-guidelines/spirit-2013-statement-defining-standard-protocol-items-for-clinical-trials/).

**Table:** 

Title {1}	Efficacy and safety of Bujing Yishi tablet for glaucoma with controlled IOP: study protocol for a multi-centre randomized controlled trial
Trial registration {2a and 2b}.	Chinese Clinical Trial Registry, ID: ChiCTR1800016431. Registered on 1 June 2018
Protocol version {3}	Date April 6, 2018 and version 2.0
Funding {4}	This trial was supported by the Chinese Medicine Bureau of Sicuan Province (2018) NO. 2018LC008.
Author details {5a}	^1.^College of Ophthalmology, Chengdu University of Traditional Chinese Medicine, Chengdu, Sichuan, 610075, China; Department of Ophthalmology, Mianyang Central Hospital, Mianyang, Sichuan, 621000, China; ^2.^Department of Ophthalmology, Hospital of Chengdu University of Traditional Chinese Medicine, Chengdu, Sichuan, 610075, China; ^3.^Department of Ophthalmology, The Affiliated Eye Hospital of Wenzhou Medical University, Wenzhou, Zhejiang, 325027, China; ^4.^Good Clinical Practice Centre, Hospital of Chengdu University of Traditional Chinese Medicine, Chengdu, Sichuan, 610075, China; ^5.^Department of Ophthalmology, Mianyang Central Hospital, Mianyang, Sichuan, 621000, China; ^6.^Department of Ophthalmology, Mianyang Hospital of Traditional Chinese Medicine, Mianyang, Sichuan, 621000, China; ^7.^Department of Ophthalmology, The Second People’s Hospital of Chengdu PiDu District, Chengdu, Sichuan, 611733, China; ^8.^Zhongshan Ophthalmic Center, Sun Yat-sen University, Guangzhou, Guangdong, 510275, China
Name and contact information for the trial sponsor {5b}	Chinese Medicine Bureau of Sicuan Province, No. 15 Yongxing lane, Jingjiang District, Chengdu, 610000, China
Role of sponsor {5c}	The study funder has no role in the study design, data collection and management, and manuscript writing publication

## Introduction

### Background and rationale {6a}

Glaucoma, affecting approximately 80 million people, is a leading cause of irreversible blindness worldwide. Degeneration of retinal ganglion cells (RGC) and progressive loss of the visual field are the primary features of glaucoma [1]. Reducing intraocular pressure (IOP) is presently the most practiced therapeutic approach for glaucoma. However, progressive loss of vision or blindness can still occur in some patients with glaucoma with controlled IOP (GPCI) [2]. It is now recognized that neuroprotective therapies are crucial in the management of glaucoma [3]. The main research on optic neuroprotective treatments have included antioxidants, N-methyl-D-aspartate (NMDA) receptor antagonists, inhibitors of glutamate release, calcium channel blockers, polyamine antagonists, nitric synthase inhibitors, and vitamin B1 [4]. Nevertheless, to date, there is no satisfying neuroprotective agent for patients with glaucoma. Gene therapy and stem cell-based applications are promising, yet they are still undergoing clinical trials. To this end, finding a satisfactory effective therapy might be a favorable way to prevent the development of glaucoma.

Traditional Chinese medicine (TCM) therapy has been used to treat glaucoma for thousands of years and has demonstrated a more favorable safety profile than conventional medicine [5, 6]. In TCM, the same disease could be diagnosed as comprising various syndromes. Therefore, the same disease will be treated differently according to the different syndromes. The syndrome of “*liver-kidney deficiency, blood stasis and fluid retention*” is an important subtype in GPCI [7, 8]. The *Bujing Yishi* tablet (BJYSP) (Good Manufacturing Practice Certificate No: 20160003HZ) is the optic nerve protector that was inherited from the legacy of Dr. Chen Dafu, who is a renowned TCM ophthalmologist in China. BJYSP consists of 10 herbs, including *salvia miltiorrhiza*, *notoginseng*, *fructus broussonetiae*, *semen cuscutae*, *schisandra chinensis*, *Chaenomeles sinensis*, motherwort fruit, *medlar*, *semen plantaginis*, and *citri reticulatae pericarpium viride*. According to TCM theory, these herbs make a concerted effect to nourish the liver and kidneys, promote blood circulation, and dredge collaterals. The therapeutic effect of BJYSP is precisely for the syndrome of “*liver-kidney deficiency, blood stasis and fluid retention*.” The safety and quality control of BJYSP is supervised by Sichuan Food and Drug Administration according to its criterion of SZBZ20070647–10(Z). We have proven many times that BJYSP protects the optic nerve from glaucoma in a glaucoma rat model; that is, BJYSP results in upregulating retinal phosphorylation-protein kinase B (P-AKT) of phosphoinositide-3 kinase (PI3K)/protein kinase B (AKT) signaling and slightly decreasing IOP [9], promoting the secretion of brain-derived neurotrophic growth factor (BDNF) and Nissl bodies [10, 11], increasing the numbers of RCGs, and improving the thickness of the retina nerve fiber layer (RNFL) and retinal ganglion cell layer (RGCL) and the ultrastructure of RCGs in a rat model of glaucoma [12].

### Objectives {7}

Although BJYSP was approved by the Sichuan Food and Drug Administration for the clinical use for hypofunction of the optic nerve and retina in 2007, it has a good effect in the treatment of patients with GPCI in the clinical setting. However, there is not enough rigorous scientific evidence to show that BJYSP is effective in treating patients with GPCI. Hence, the aim of the present study was to clarify the visual function protective effects of BJYSP by comparing it with a positive control drug (mecobalamin tablet) in patients with GPCI.

### Trial design {8}

The study is designed as a 6-month, multicenter, stratified trial following the prospective, randomized, open-label, blinded endpoint (PROBE) study. The purpose is to evaluate the safety and effect of BJYSP in patients with GPCI. The clinical study was approved by the Sichuan Regional Ethics Review Committee on Chinese Medicine and Medical Ethics Committee of Affiliated Hospital of Chengdu University of TCM (approval no. 2018KL-040). The study was registered at the Chinese Clinical Trial Registry (ChiCTR1800016431, website: http://www.chictr.org.cn). The study will be strictly conducted according to the principles of the Declaration of Helsinki as well as Good Clinical Practice (GCP) guidelines. The trial will be completed according to the Standard Protocols Items: Recommendations for Intervention Trials (SPIRIT) Checklist.

## Methods: participants, interventions, and outcomes

### Study setting {9}

**Fig. 1:**
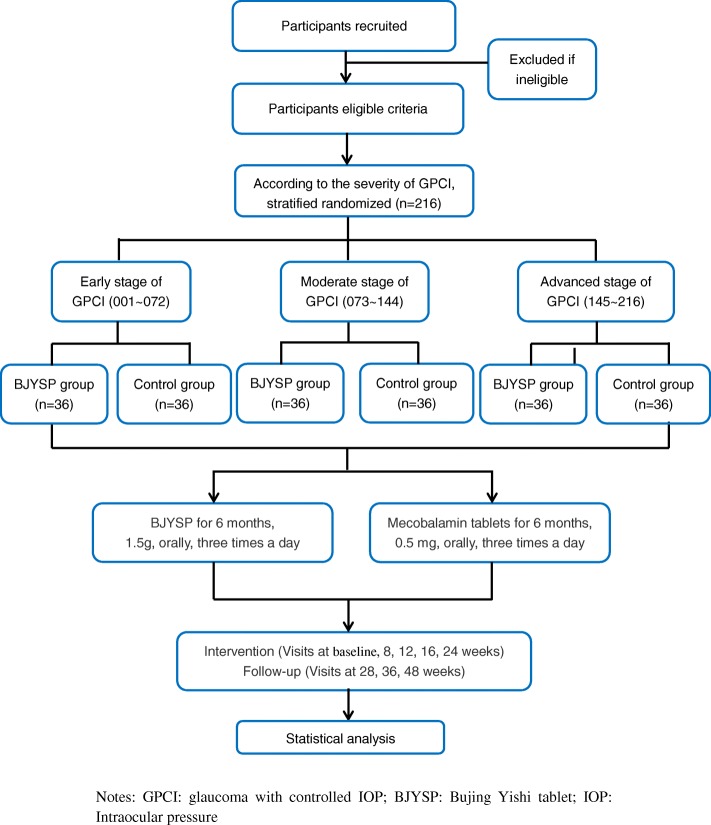
Flow chart of clinical trial in patients with GPCI. BJYSP Bujing Yishi tablet, GPCI glaucoma with controlled IOP, IOP intraocular pressure

Potential participants with GPCI will be recruited from the Hospital of Chengdu University of TCM (Sichuan, China), MianYang Hospital of TCM (Sichuan, China), and The Second People’s Hospital of Chengdu PiDu District (Sichuan, China). Our research will use the Internet, hospital announcements, and posters to provide a hotline to call for potential volunteers. If the patients are interested in our study, they will be told of the purpose and content of research as well as the benefits and drawbacks of participating in detail. After screening, the participants meeting the inclusion criteria will be enrolled. The study flow is shown in in the SPIRIT Figure (Fig. 1). Participants will undergo a 6-month treatment period and a 6-month follow-up period.

### Eligibility criteria {10}

#### Inclusion criteria

All eligible participants should meet the following criteria: (1) diagnosed with primary open-angle glaucoma (POAG) [13]: pathologic high intraocular pressure (HIOP; 24 h peak IOP > 21 mmHg (1 mmHg = 0.133 kPa), characteristic glaucomatous neuropathy (RNFL defect or optic disc change), and/or visual field loss, open anterior chamber angle, exclusion of other causes result in HIOP, only one eye from each patient will be chosen; (2) have a visual field defect that could be evaluated by standard automated perimetry (reliability factor < 15%) and an IOP that is < 18 mmHg (at least 3 months after anti-glaucoma surgery); (3) meet the syndrome pattern of kidney deficiency and blood stasis; (4) withdraw from using other optic nerve protective agents for > 2 months; (5) have spherical refractive error between + 3.00 and − 6.00 diopters, clearly visible fundus without pupil dilation, and best-corrected visual acuity (BCVA) ≥ 0.3; (6) be aged 18–75 years; (7) be in a conscious status and able to cooperate with the examination and treatment; and (8) be willing to take part in the study and sign informed consent.

#### Exclusion criteria

Participants with any one of the following will be excluded: (1) poorly controlled IOP (≤ 7 mmHg or > 18 mmHg) accompanied by thin filtering cystic bleb or bleb leakage; (2) glaucoma other than open-angle glaucoma; (3) a syndrome pattern that does not meet the criteria of kidney deficiency and blood stasis; (4) other macular degeneration, cataract, proliferative diabetic retinopathy, and retinal vascular occlusion or other diseases that might cause visual field loss; (5) pregnancy or lactation; (6) abnormal severe primary hepatic or renal or serious systemic diseases such as heart diseases, primary hypertension, diabetes mellitus, or gastric ulcer; or (7) an allergic history to the ingredients of BJYSP or mecobalamin tablets.

#### TCM syndrome differentiation

The TCM syndrome of kidney deficiency and blood stasis will be based on guidelines delineated in the *Clinical Research of New Investigational Drugs in Traditional Chinese Medicine* [14]. The diagnostic standards are as follows: primary signs and symptoms include blurry visual acuity, narrow visual field, and eye distension; and secondary signs and symptoms include dry eye, limp aching lumbus and knees.

For the diagnosis of GPCI, participants will be diagnosed with both kidney deficiency and blood stasis syndrome under the condition of meeting at least two of the primary signs/symptoms and two or more secondary signs/symptoms, and the necessary condition is dark purple tongue and thready pulse with deep, or string, or unsmooth. The investigators will be required to be trained in standard operating procedures (SOPs) of scrutinization of TCM symptoms and administer a symptom assessment survey to every participant according to TCM Symptom Score Scale (Table 1).

**Table 1:** Traditional Chinese medicine (TCM) symptom score scale

Blurry visual acuity	▫ 0: None
	▫ 2: Mild blurred visual acuity
	▫ 4: Unable to read
	▫ 6: Difficult to see anything
Narrow visual field	▫ 0: None
	▫ 2: As if something obscures visual acuity
	▫ 4: Inconvenient to walk
	▫ 6: Difficult to walk
Eye distension	▫ 0: None
	▫ 2: Mild eye distension
	▫ 4: Endurable eye distension
	▫ 6: Intolerable eye distension
Dry eye	▫ 0: None
	▫ 2: Mild dry eye
	▫ 4: Obvious dry eye, as if there is a foreign body in it
	▫ 6: Intolerable dry eye, frequent blink
Limp aching lumbus and knees	▫ 0: None
	▫ 2: Early morning aching lumbus, which can be alleviated by beating; mild limp knees
	▫ 4: Continuous aching lumbus, which will be aggravated when working knees are too limp to carry heavy weight
	▫ 6: Severe aching lumbus, which will not be relieved by rest; knees are too limp to desire to walk
Dark purple tongue	▫ 0: No
	▫ 2: Yes
Thready pulse deep, string or unsmooth	▫ 0: No
	▫ 2: Yes

### Who will take informed consent? {26a}

Linru Cui, Chao Wang, and Zhao Xie will obtain informed consent from potential trial participants at each site, respectively.

### Additional consent provisions for collection and use of participant data and biological specimens {26b}

Not applicable.

### Interventions

#### Explanation for the choice of comparators {6b}

In this trial, we will investigate whether BJYSP can effectively protect the optic nerve and improve visual function for GPCI. The mecobalamin tablets are currently being used as a routine treatment for treating visual function impairment of GPCI [15, 16]. Therefore, mecobalamin was chosen as a positive-control medicine in our trial. In addition, to observe the effect of BJYSP on GPCI with different disease severity, GPCI will be classified as early, moderate, and advanced based on the visual field defects of patients.

#### Intervention description {11a}

Participants will receive BJYSP or mecobalamin tablets for 6 months. The BJYSP (0.3 g/ tablet × 5) and mecobalamin tablets (0.5 mg/ tablet × 1) will be orally administered three times a day for 6 months in the BJYSP group and control group, respectively. BJYSP is provided by the Teaching Hospital of Chengdu University of TCM, Chengdu, China (Good Manufacturing Practice Certificate No: 20160003HZ). BJYSP consists of 10 herbs, including *salvia miltiorrhiza*, *notoginseng*, *fructus broussonetiae*, *semen cuscutae*, *schisandra chinensis*, Chaenomeles sinensis, motherwort fruit, *medlar*, *semen plantaginis*, and *citri reticulatae pericarpium viride*. According to TCM theory, these herbs make a concerted effect to nourish the liver and kidneys, promote blood circulation, and dredge collaterals. The safety and quality control of BJYSP is supervised by Sichuan Food and Drug Administration according to its criterion of SZBZ20070647–10 (Z). Mecobalamin tablets are provided by North China Pharmaceutical Co. Ltd., Shijiazhuang, China (Approval no. H20031126). Participants will be advised to refrain from other treatments during the study. The study will also include IOP-control medicine without the effect of optic nerve protection such as β-adrenergic blockers, adrenergic agonists, and carbonic anhydrase inhibitors.

#### Criteria for discontinuing or modifying allocated interventions {11b}

Participants will be free to withdraw from the clinical study and choose other treatments (e.g. citicoline sodium tablets) if they feel their disease will not improve. Reasons for discontinuation of treatment may include but are not limited to, the following reasons: (1) participant becomes pregnant; (2) participant experiences serious side effects or complications; (3) participant takes other medicine during the study; (4) participant does not comply with the study protocol; and (5) participant requests withdrawal for other reasons.

#### Strategies to improve adherence to interventions {11c}

Frequent follow-up phone calls are an important aspect for monitoring adherence.

#### Relevant concomitant care permitted or prohibited during the trial {11d}

Participants will be informed and educated to refrain from all other therapies to improve visual function during the trial. IOP-control medications without the effect of optic nerve protection such as β-adrenergic blockers, adrenergic agonists, and carbonic anhydrase inhibitors are permitted.

### Provisions for post-trial care {30}

Not applicable.

#### Outcomes {12}

##### Primary outcomes

Primary outcomes help us to determine whether visual function is improved and whether BJYSP is effective. They will be as follows: (1) mean change in BCVA in the study eye will be measured at baseline and 2, 4, and 6 months thereafter; (2) mean change in values of the pattern standard deviation (PSD), mean defect (MD), square root of loss variance (sLV) in the study eye, as determined by an Octopus 900 perimeter, at baseline and 2, 4, and 6 months thereafter; (3) mean change in values of the latency of negative response 75 (LN75), amplitude of negative response 75 (AN75), latency of positive response 100 (LP100), and amplitude of positive response 100 (AP100) in the study eye, as determined by VTAS 2000 VEP, at baseline and 2, 4, and 6 months thereafter; and (4) mean change in values of the disk area (DA), cup area (CA), rim area (RA), cup volume (CV), rim volume (RV), cup/disk area ratio (C/DAR), linear cup/disk ratio (C/DLR), mean cup depth (mCD), maximum cup depth (maxCD), cup shape measure (CSM), height variation contour (HVC), mean RNFL thickness (MRNFLT), and RNFL cross-sectional area (RCSA) in the study eye, as determined by Heidelberg retina tomography II (HRT II), at baseline and 2, 4, and 6 months thereafter.

**Fig. 2:**
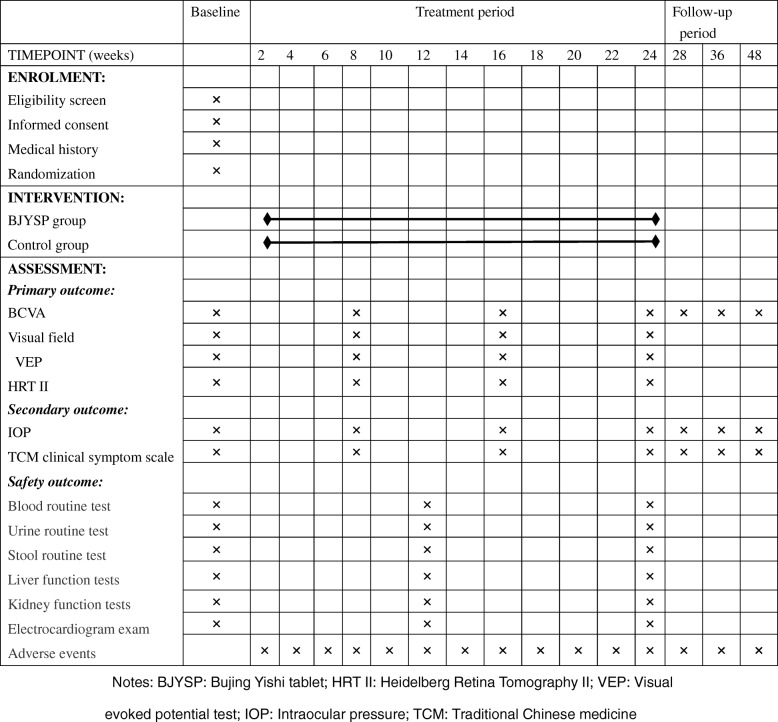
Measurement items and points of data capture. BJYSP Bujing Yishi tablet, HRT II Heidelberg Retina Tomography II, IOP intraocular pressure, TCM Traditional Chinese medicine, VEP visual evoked potential

Potential participants will be told in detail of the purpose and content of research as well as the benefits and drawbacks of participating. Informed consent will be obtained by the designee at each site from those willing to participate. The schedule of visits and tests is shown in the SPIRIT Figure (Fig. 2). Participants will be reminded by telephone the week before the study visit by research nurses at each site.

##### Secondary outcomes

Secondary outcomes help us to know if the BJYSP improves TCM clinical symptoms and the changes in IOP: (1) mean change in IOP in the study eye, as determined by TOPCON CT 60 non-contact tonometer, at baseline and 2, 4, and 6 months thereafter; and (2) change in the number of patients with different grades of TCM symptoms, as determined by the investigators trained in SOPs according to TCM Symptom Score Scale (Table 1), at baseline and 2, 4, and 6 months thereafter.

##### Safety assessment

Safety assessments by three routine tests (blood, urine, and stool), electrocardiogram, liver function (alanine aminotransferase [ALT], aspartate aminotransferase [AST], alkaline phosphatase [ALP], gamma-glutamyl transferase [GGT], and serum total bilirubin [TBIL]) and kidney function (blood urea nitrogen [BUN], SCr, uric acid [UA], and β2-microglobulin) will be measured at baseline and 3 and 6 months thereafter.

### Participant timeline {13}

#### Sample size {14}

The calculation of the sample size is based on previous studies. The effective rate of mecobalamin tablets for GPCI was approximately 85% [17, 18], We hypothesized a 90% effective rate of BJYSP for GPCI in a larger sample size. According to the sample size of the estimation formula [19], α = 0.05, β = 0.10, in a two-sided test, P1 = 0.67, P2 = 0.871, two-sided u_α/2_ = u_0.05/2_ = 1.96, one-tailed u_β_ = u_0.1_ = 1.282. We put these values into the formula n^1^ = n^2^= $\frac{1}{2}{\left[\frac{{\mathrm{u}}_{\upalpha /2}+{\mathrm{u}}_{\upbeta}\ }{\sin^{-1}\sqrt{p_1}-{\sin}^{-1}\sqrt{p_2}}\right]}^2$, resulting in n^1^ = n^2^= $\frac{1}{2}{\left[\frac{1.96+1.282\ }{\sin^{-1}\sqrt{0.67}-{\sin}^{-1}\sqrt{0.871}}\right]}^2=87.86.$ That is, approximately 88 cases in the BJYSP group and in the control group or 176 cases in both groups. To account for a 20% dropout rate, the calculation 176 + (176 × 20%) = 211.2 suggests that 212 patients should be recruited. We rounded this value to 216 patients in total.

#### Recruitment {15}

Potential participants with GPCI will be recruited from the Hospital of Chengdu University of TCM (Sichuan, China), MianYang Hospital of TCM (Sichuan, China) and The Second People’s Hospital of Chengdu PiDu District (Sichuan, China). Our research will use the Internet, hospital announcements, and posters to provide a hotline to call for potential volunteers. If the patients are interested in our study, they will be told of the purpose and content of research as well as the benefits and drawbacks of participating in detail. After screening, the participants meeting the inclusion criteria will be enrolled. The study flow is shown in in the SPIRIT Figure (Fig. 1). Participants will undergo a 6-month treatment period and a 6-month follow-up.

### Assignment of interventions: allocation

#### Sequence generation {16a}

After obtaining the written informed consent from the participants and baseline screening, all eligible patients will be stratified according to early, moderate, and advanced stages of glaucoma [20]: early glaucoma patients have an early glaucomatous visual field loss defined as an MD ≥ − 6 dB; moderate glaucoma patients have a moderate glaucomatous visual field loss defined as MD ≤ − 6 to ≥ − 12 dB; and advanced glaucoma patients had an advanced glaucomatous visual field loss defined as MD ≤ − 12 to ≥ − 20 dB. Random numbers of 001–216 will be automatically generated by SAS software (whole random numbers). Among them, the random numbers 001–072 are assigned to the early stage, the random numbers 073–144 are assigned to the moderate stage, and the random numbers 145–216 are assigned to the advanced stage. After stratifying, the participants will be randomly assigned to the BJYSP group or control group with a ratio of 1:1. An overview of case distribution of each center, specific measurements, and time points of data collection can be found in the SPIRIT Figure (Fig. 2).

#### Concealment mechanism {16b}

Random numbers will be placed in a same shape and size envelope and subsequently sealed for concealing group allocation and to avoid selection bias. The allocation random sequence will be stored and concealed from the investigators, statistician, and outcomes assessors to prevent detection bias.

#### Implementation {16c}

The designated statisticians will generate the allocation sequence, different investigators designated by the project leader will enroll or assign participants.

### Assignment of interventions: blinding

#### Who will be blinded {17a}

In this PROBE design trial, both participants and treating clinicians will be aware of the treatment assignment. Information including BCVA, visual field, VEP, HRT II, IOP, and TCM clinical symptom scales will be assessed by independent assessors who are blind to the assignment and treatment. The principal investigators, statistician, and outcomes assessors will be blind to the treatment assignments until the database is locked.

#### Procedure for unblinding if needed {17b}

Unblinding the investigators will be permissible only in specific situations, such as when knowledge of the actual treatment is highly necessary for the appropriate management of participants (e.g. serious adverse events).

### Data collection and management

#### Plans for assessment and collection of outcomes {18a}

An overview of specific measurements and time points for data collection can be found in the SPIRIT Figure. All data will be recorded in detail in the case report form (CRF) after face-to-face visits or telephone calls to the participants. The same investigator will be responsible for the examination of each patient at each assessment. Baseline measurements, demographics, medical history, and previous medications will be collected at the first visit only. Primary and secondary outcomes and safety indicators will be collected every 8 weeks from baseline until week 24. After training in advance and passing a test, two data entry clerks will recheck the CRF and then independently perform data entry. The statistical package Epidata 3.1 will be used for data entry. The data will then be transferred to the statistical SPSS 21.0 software (SPSS Inc., Chicago, IL, USA) for data analysis.

#### Plans to promote participant retention and complete follow-up {18b}

Frequent follow-up phone call will be conducted to promote participant retention.

#### Data management {19}

After training in advance and passing a test, two data entry clerks will recheck the CRF and then independently perform data entry back to back. The statistical package Epidata 3.1 will be used to check entry consistency and seek out abnormal data in accordance with a pre-set value range automatically. The third person (data manager) independently checks and judges the input data. The principle investigators from each of the trial sites will confirm whether all CRFs have been timely completed, ensure that the withdrawal of participants and all adverse events are documented in CRFs, and so on). All original data and relevant records will be properly classified and stored at each study center under confidential conditions for 3 years.

#### Confidentiality {27}

Participants’ personal information will be kept confidential in the same way as their medical histories in the hospital before, during, and after the trial.

#### Plans for collection, laboratory evaluation, and storage of biological specimens for genetic or molecular analysis in this trial/future use {33}

Not applicable.

### Statistical methods

#### Statistical methods for primary and secondary outcomes {20a}

Statisticians will independently undertake statistical analysis. All data will be presented as the means ± standard deviation (SD). Statistical analysis will be performed by SPSS 21.0 software (SPSS Inc., Chicago, IL, USA). All statistical tests will be two-sided tests, and the statistical significance threshold will be set at *P* < 0.05. The chi-square test will be used for categorical variables, an independent Student’s t-test will be used on measurement data, and rank-sum test will be used for grade data. To assess covariate balance, baseline characteristics will be summarized and compared between the BJYSP group and the control group by means of simple descriptive statistics.

#### Interim analyses {21b}

Not applicable.

#### Methods for additional analyses (e.g. subgroup analyses) {20b}

Not applicable.

#### Methods in analysis to handle protocol non-adherence and any statistical methods to handle missing data {20c}

Due to the anticipated small number of missing data, we will dispose missing data by transfer the last data to the final data. The data of this participant will be entered into the Full Analysis Set (FAS) in an invalid way, and this case will not be entered into the Per Protocol Set (PPS). Finally, the statistical significance of FAS and PPS will be analyzed. If the statistical significance was consistent, there was statistical difference.

#### Plans to give access to the full protocol, participant-level data, and statistical code {31c}

No plans.

### Oversight and monitoring

#### Composition of the coordinating center and trial Steering Committee {5d}

The Steering Committee (SC) will be composed of the subject leader and the project manager. The SC responsible for the management of the whole project.

The inspectors of the Monitor Group (MG) are appointed by the SC. The MG monitored the whole process of the study in accordance with the GCP specification. The inspector monitors the investigator’s compliance with the protocol, protection of participants’ rights and interests, the quality of the CRF form, and the investigators’ knowledge of various standards and then writes the inspection reports to the SC.

#### Composition of the data monitoring committee, its role, and reporting structure {21a}

No data monitoring committee has been invited for this study because of the expected low incidence of adverse events and the low numbers in each center. Statistical package Epidata 3.1 will be used to build the database, routine data monitoring will be performed according to the standard operating procedures of sponsor. The trial is managed by the SC and with oversight from the MG.

#### Adverse event reporting and harms {22}

Adverse events, such as signs and symptoms and other discomforts, will be observed and recorded in detail during the study and follow-up phases. All adverse events will be strictly recorded, monitored, and treated until resolved.

#### Frequency and plans for auditing trial conduct {23}

Our trials will be audited every 6 months by the supervisor who has successfully completed pharmaceutical clinical trial and the review process will be independent of investigators and sponsors.

#### Plans for communicating important protocol amendments to relevant parties (e.g. trial participants, ethical committees) {25}

If there are changes to eligibility criteria, outcomes, and analyses, a new version of protocol will be submitted to the Medical Ethics Committee of the Affiliated Hospital of Chengdu University of Traditional Chinese Medicine for approval.

#### Dissemination plans {31a}

The results of the research will be presented in the form of publication or conference reports.

## Discussion

As the leading cause of irreversible blindness, glaucoma brings a huge burden to patients, families, and society. Therefore, developing medicines that protect visual function in patients with glaucoma has become a hotspot for public health [21]. Reducing IOP and neuroprotective treatments are presently the most practiced visual function protective therapeutic approach for glaucoma. However, progressive loss of vision or blindness can still occur in some patients with GPCI, and the neuroprotection for glaucoma remains unsatisfactory at present [22]. Therefore, exploring an effective neuroprotective agent for patients with glaucoma is important and urgent.

TCM harbors a long history and a unique advantage in treating neurodegenerative diseases, such as glaucoma [23]. BJYSP has been widely used in the treatment of GPCI, but useful empirical research is insufficient for its popularization and application. The disadvantages of less rigorous designs, small sample sizes, and low quality have led to many TCM studies being insufficient to convince people of its value.

In this trial, we will investigate whether BJYSP can effectively protect the optic nerve and improve visual function for GPCI. The mecobalamin tablets are currently being used as a routine treatment for treating visual function impairment of GPCI [15, 16]. Therefore, mecobalamin was chosen as a positive-control medicine in our trial. In addition, to observe the effect of BJYSP on GPCI with different disease severity, GPCI will be classified as early, moderate, and advanced based on the visual field defects of patients.

The PROBE design is not a double-blind, placebo-controlled trial, but the assessors, statisticians, and investigators who perform each examination on patients will be completely blinded to group allocation. In addition, a double-blind, placebo-controlled design would cause considerably higher costs compared with the PROBE design. Furthermore, the potential advantage of the PROBE design is that the effect of BJYSP will resemble the effect in clinical practice, and the PROBE design has been extensively proven to yield the same results as placebo-controlled designs [24, 25]. Nevertheless, to guarantee the quality of this study, the design and execution of the study will be strictly performed with proper quality control. A training session for each center will be held to explain the detailed study protocol, the diagnosis of the TCM syndrome pattern, and the SOPs. The same investigator will be responsible for the examination of each patient at different time periods. All indicators will be assessed by independent assessors. The principal investigators, statistician, and outcomes assessors will be blind to the treatment assignments until the database was locked.

In conclusion, the purpose of this research is to verify the therapeutic efficacy and safety of BJYSP. The outcome of the study will provide evidence-based data for the use of BJYSP to treat GPCI with liver-kidney deficiency, blood stasis, and fluid retention syndrome, thereby providing a new avenue in the treatment of GPCI.

### Trial status

Recruitment began in June 2018 and the approximate date when recruitment will be completed is April 2020. Protocol version 2.0 was approved on 6 April 2018.

## Data Availability

Not applicable.

## Abbreviations

GPCI: Glaucoma with controlled IOP
BJYSP: Bujing Yishi tablet
PROBE: Prospective, randomized, open-label, blinded endpoint
BCVA: Best-corrected visual acuity
VEP: Visual evoked potential
HRT II: Heidelberg Retina Tomography II
IOP: Intraocular pressure
TCM: Traditional Chinese medicine
RGCs: Retinal ganglion cells
RNFL: Retina nerve fiber layer
RGCL: Retinal ganglion cells layer;
GCP: Good Clinical Practice
SOPs: Standard operating procedures
AEs: Adverse events
CRF: Case report form
